# Structural dynamics of the yeast Shwachman-Diamond syndrome protein (Sdo1) on the ribosome and its implication in the 60S subunit maturation

**DOI:** 10.1007/s13238-015-0242-5

**Published:** 2016-02-05

**Authors:** Chengying Ma, Kaige Yan, Dan Tan, Ningning Li, Yixiao Zhang, Yi Yuan, Zhifei Li, Meng-Qiu Dong, Jianlin Lei, Ning Gao

**Affiliations:** School of Life Sciences, Tsinghua University, Beijing, 100084 China; National Institute of Biological Sciences, Beijing, 102206 China; Graduate Program in Chinese Academy of Medical Sciences and Peking Union Medical College, Beijing, 100730 China

**Keywords:** ribosome biogenesis, SBDS, SDS, Sdo1, cryo-electron microscopy (cryo-EM)

## Abstract

The human Shwachman-Diamond syndrome (SDS) is an autosomal recessive disease caused by mutations in a highly conserved ribosome assembly factor SBDS. The functional role of SBDS is to cooperate with another assembly factor, elongation factor 1-like (Efl1), to promote the release of eukaryotic initiation factor 6 (eIF6) from the late-stage cytoplasmic 60S precursors. In the present work, we characterized, both biochemically and structurally, the interaction between the 60S subunit and SBDS protein (Sdo1p) from yeast. Our data show that Sdo1p interacts tightly with the mature 60S subunit in vitro through its domain I and II, and is capable of bridging two 60S subunits to form a stable 2:2 dimer. Structural analysis indicates that Sdo1p bind to the ribosomal P-site, in the proximity of uL16 and uL5, and with direct contact to H69 and H38. The dynamic nature of Sdo1p on the 60S subunit, together with its strategic binding position, suggests a surveillance role of Sdo1p in monitoring the conformational maturation of the ribosomal P-site. Altogether, our data support a conformational signal-relay cascade during late-stage 60S maturation, involving uL16, Sdo1p, and Efl1p, which interrogates the functional P-site to control the departure of the anti-association factor eIF6.

**Electronic supplementary material:**

The online version of this article (doi:10.1007/s13238-015-0242-5) contains supplementary material, which is available to authorized users.

## Introduction

Protein biosynthesis is catalyzed by the ribosome in all living organisms. Thousands of ribosomes must be synthesized per minute to maintain and update the tremendous protein inventory in rapidly growing cells. Ribosomal subunit assembly *in vivo* is a highly complex process, which composes of a large number of intertwined ribosomal protein (RP) binding and rRNA maturation (rRNA folding, processing, modification and assembly) events. In eukaryotes, additional complexity is added, as the assembly starts in the nucleolus and involves the import of RPs and associated factors, as well as the export of premature ribosomal particles (66S and 43S pre-ribosomes) across nuclear membrane. Final maturation of the pre-ribosomes takes place in cytoplasm, and is coupled to the regulation of translation initiation (Karbstein, 2013; Lebaron et al., 2012; Miluzio et al., 2009; Soudet et al., 2010; Strunk et al., 2012). In *Saccharomyces cerevisiae*, the complete maturation of translationally active 60S and 40S subunits from extremely long rRNA transcripts and 79 RPs requires more than 70 small nucleolar RNAs (snoRNAs) and 200 assembly factors (AFs) (Panse and Johnson, 2010; Woolford and Baserga, 2013). Many of these factors function primarily on the quality control of subunit production, acting at various maturation checkpoints during the assembly process. These checkpoints often involve regulated binding and release of a series of factors (Karbstein, 2013; Lo et al., 2010; Matsuo et al., 2014).

Cellular defects in ribosome biogenesis caused by assembly factor insufficiency or mutations result in arrests of assembly at different checkpoints during cell cycle progression (Bernstein et al., 2007; Dez and Tollervey, 2004; Jorgensen et al., 2002). More importantly, disorders in ribosome biogenesis, which induce a nucleolar stress (Boulon et al., 2010) that is monitored by the Mdm2/Hdm2-p53 pathway (Chakraborty et al., 2011; Deisenroth and Zhang, 2010), were shown to be associated with increased cancer susceptibility in animal cells (Montanaro et al., 2008; Ruggero and Pandolfi, 2003). In human, a diverse collection of genetic diseases, named as ribosomopathies, have been linked to mutations in ribosomal proteins or AFs (Chakraborty et al., 2011; Freed et al., 2010; Narla and Ebert, 2010; Teng et al., 2013). Besides their specific clinical phenotypes, patients with ribosomopathies have a predisposition to a variety of cancers. Among these diseases, Shwachman-Diamond syndrome (SDS) is an autosomal recessive disease with multi-system disorders caused by mutations in the highly conserved Shwachman-Bodian-Diamond Syndrome gene (SBDS) (Boocock et al., 2003). Clinical characteristics associated with SDS are pancreatic insufficiency, skeletal abnormalities and bone marrow failure with neutropenia, ineffective hematopoiesis, and increased risk of leukemia (Narla and Ebert, 2010). Most of SDS patients (~90%) are associated with mutations of *SBDS* gene that result in premature truncation of SBDS protein (Austin et al., 2005; Boocock et al., 2003).

SBDS is a highly conserved protein in archaea and eukaryotes (Boocock et al., 2006; Shammas et al., 2005). Converging cell biology data on several SBDS homologues, including yeast (Sdo1p) (Lo et al., 2010; Luz et al., 2009; Menne et al., 2007; Moore et al., 2010; Savchenko et al., 2005), mouse (Finch et al., 2011), *Dictyostelium discoideum* (Wong et al., 2011) and human SDS patient cells (Burwick et al., 2012; Ganapathi et al., 2007; Wong et al., 2011) have implicated a functional role of SBDS in the maturation of the 60S ribosomal subunit. Specifically, SBDS was proposed to coordinate with elongation factor-like 1 (Efl1p) to release eIF6 (Tif6p in yeast), an important 60S shuttling factor, from late cytoplasmic pre-60S particles. Failure in the timely release and recycling of Tif6p impairs the subunit joining and subsequent translation initiation (Karbstein, 2013; Miluzio et al., 2009). The structures of SBDS from several species have been resolved (de Oliveira et al., 2010; Finch et al., 2011; Ng et al., 2009; Shammas et al., 2005), which contain three structural domains, I to III (numbered from the N-terminus). The N-terminal domain of SBDS was shown to be involved in RNA binding (de Oliveira et al., 2010) and domains II-III were found to interact with an insertion domain of Efl1p (Asano et al., 2014). Also, recent data revealed a functional link between Sdo1p and uL16 (RPL10), which is a late-binding protein during 60S assembly (Gamalinda et al., 2014). It was shown that the loop of uL16 residing in the ribosomal P-site is important for the activation of Efl1p to induce the release of Tif6p (Bussiere et al., 2012). Furthermore, uL16 was shown to be involved in the recruitment of Sdo1p (Sulima et al., 2014a), and a role of Sdo1p/SBDS as a nucleotide exchange factor to stabilize the binding of GTP to Efl1p was proposed (Gijsbers et al., 2013).

Despite the functional framework that defines a pathway for SBDS protein family as described above, its biochemical property that contributes to its involvement in the cytoplasmic recycling of Tif6p and ribosomal P-site maturation remains unclear. In this report, using cryo-electron microscopy (cryo-EM) and several complementary approaches, we performed structural and biochemical characterization of the interaction between the yeast SBDS homologue, Sdo1p and the 60S subunit. Our data reveal that Sdo1p binds to the ribosomal P-site and directly contacts H69 and H38 of the 25S rRNA. Moreover, owing to its structural flexibility, Sdo1p displays a dynamic behavior on the 60S subunit, with wobbling terminal domains (II and III). Very interestingly, Sdo1p is able to induce dimerization of the 60S subunits in a very specific manner. Together with published data, our results suggest that Sdo1p is an essential surveillance factor for the 60S maturation, which monitors the conformational status of the ribosomal P-site, including surrounding uL16, H69 and H38, and couples, through Efl1p, the maturation of the ribosomal P-site to the release of Tif6p.

## RESULTS

### Sdo1p binds to the mature 60S subunit *in vitro*

**Figure 1:**
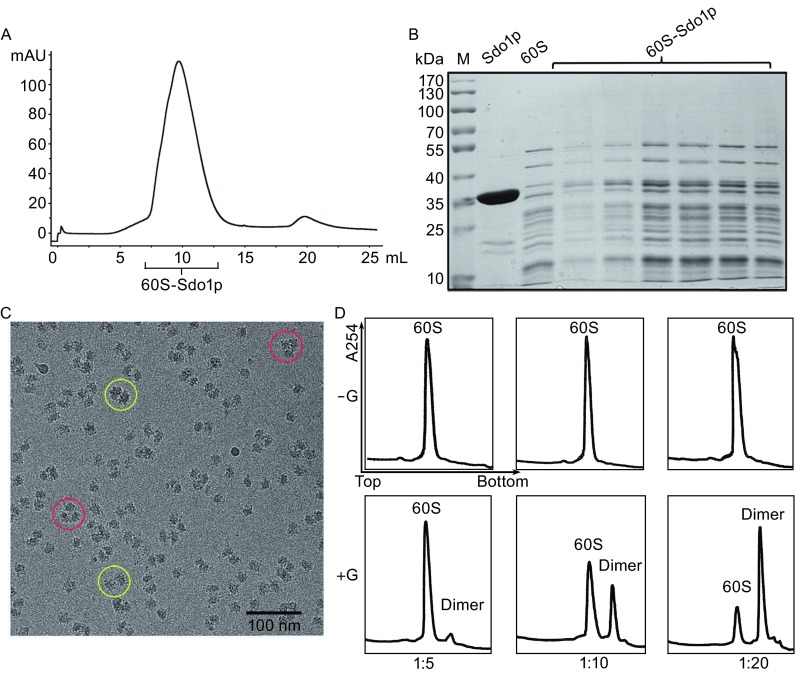
Sdo1p binds to the mature 60S subunit *in vitro*. (A) Gel filtration trace of the 60S-Sdo1p complex monitored by UV absorption at 280 nm. (B) SDS-PAGE examination of fractions labelled in (A). M, maker lane; Sdo1p, loading input containing Sdo1p only; 60S, loading input containing 60S only. (C) A representative cryo-EM micrograph of the 60S-Sdo1p complex (without crosslinking). Particles of 60S dimers and trimers are highlighted by yellow and red circles, respectively. (D) Examination of the formation of 60S dimers by Sdo1p using sucrose density gradient centrifugation. 5-, 10- or 20-fold excess of Sdo1p was incubated with the 60S subunit in the absence (−G) or presence (+G) of 0.1% glutaraldehyde

Previous data showed that Sdo1p co-fractionated with the pre-60S fractions (Menne et al., 2007) and bound to all forms of rRNA *in vitro* (Luz et al., 2009). It is not clear whether Sdo1p could bind to the mature form of the 60S subunit. This prompted us to examine the interaction between the mature 60S subunit and Sdo1p by gel filtration analysis. As shown in Fig. 1A and 1B, Sdo1p co-elutes with the 60S subunit, and forms a stable complex with the mature 60S subunit.

### Sdo1p induces dimerization of 60S subunits *in vitro*

Next, we applied cryo-EM to analyze the 60S complex bound with Sdo1p, aiming at determining the Sdo1p binding site in a minimal system. The resulting images of the 60S-Sdo1p complex show a relatively even distribution, without undesired aggregation or precipitation (Fig. 1C). However, unexpectedly, defined oligomers of 60S subunits were also detected. In addition to well separated mono-disperse 60S monomers, many 60S subunits were found to exist in a dimeric or trimeric state (Fig. 1C). The gel filtration analysis does not have enough resolution at its high molecular weight boundary, therefore, we employed sucrose density gradient centrifugation (SDGC) to confirm this observation. However, in contrast to the cryo-EM results, there were no apparent changes in the sedimentation profiles of the 60S subunit in the presence of 5-, 10- and 20-fold excess of Sdo1p (Fig. 1D, upper panel). We reason that the seemingly discrepancy between the two approaches might be explained by the weak association between the two 60S subunits which might not sustain a long-time centrifugation. Indeed, upon addition of a chemical crosslinker (0.1% glutaraldehyde) in the reaction system, an additional peak, corresponding to the 60S dimer, appears on the gradient profile. Dimerization of 60S subunits by Sdo1p is clearly concentration dependent: in 5-fold excess of Sdo1p, most of the 60S subunits are still in monomeric state; in 10-fold excess, around 40% of 60S subunits are dimers; in 20-fold excess, the majority of the 60S subunits are in dimer, and tiny peaks corresponding to higher order oligomers start to emerge (Fig. 1D, lower panel). And the empty 60S subunits could not form dimer with 0.1% glutaraldehyde (Fig. S1).

### Sdo1p does not induce oligomerization of 40S or 80S ribosomes

**Figure 2:**
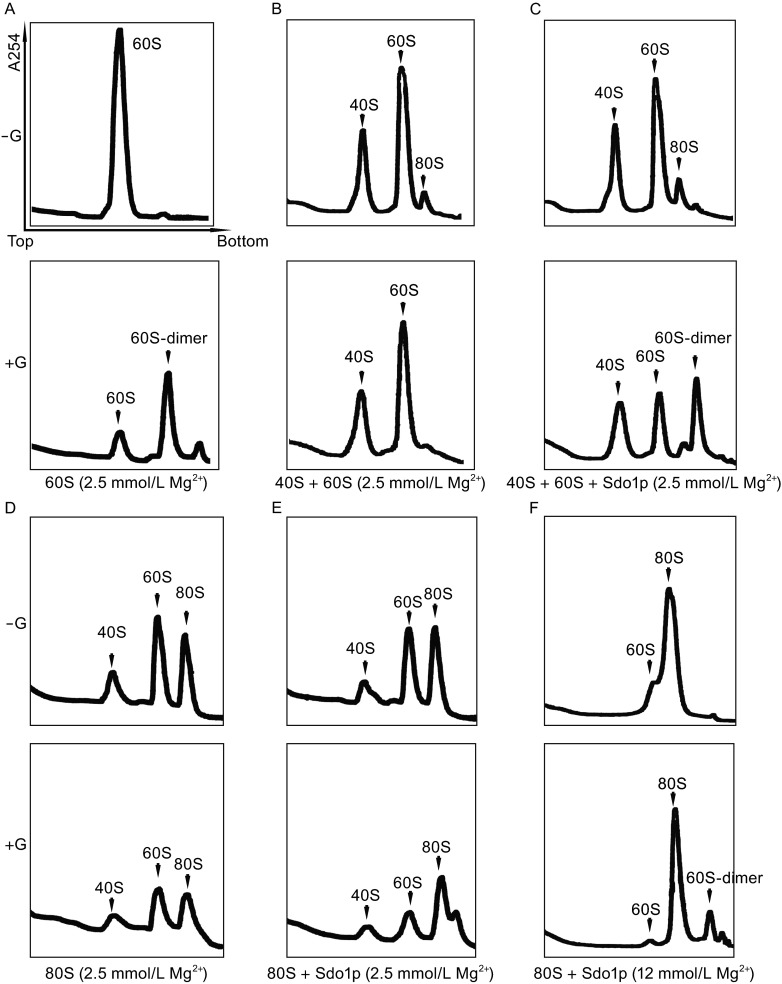
Sdo1p only specifically induces 60S dimerization but not 40S or 80S ribosome. (A) Sdo1p induces the formation of dimers of 60S subunits. (B) A mixture of the 40S and 60S subunits in dissociating condition (2.5 mmol/L Mg^2+^), in the absence (−G, top panel) or presence (+G, bottom panel) of 0.1% glutaraldehyde, were subjected to SDGC, indicating that glutaraldehyde alone has no effect on dimer formation. (C) Same as (B), but with 20-fold excess of Sdo1p supplied. (D) 80S ribosomes in dissociating condition, showing a partial dissociation of 80S into 40S and 60S subunits. (E) Same as (D), but with 20-fold excess of Sdo1p supplied indicated that Sdo1p do not induce 40S and 80S ribosome dimerization but only 60S. (F) 80S ribosomes in associating condition (12 mmol/L Mg^2+^), treated with Sdo1p in the absence or presence of glutaraldehyde also showed Sdo1p do not induce 80S ribosome dimerization

Following above observations, we tested whether Sdo1p had any effects on the sedimentation profiles of a mixture of 40S and 60S subunits (2.5 mmol/L Mg^2+^) or 80S ribosomes (12 mmol/L Mg^2+^) using SDGC. As shown in Fig. 2, addition of Sdo1 has no effect on the 40S or the 80S ribosome, except that it again preferentially dimerizes the 60S subunits in the reaction mixtures. And the effect is more apparent in the presence of glutaraldehyde and when the free 60S subunit is abundant (Fig. 2C, lower panel). Due to the equilibrium of 80S association and dissociation, there would be a certain amount free 60S subunit in the 80S mixture even in the associating condition (12 mmol/L Mg^2+^), and as expected, Sdo1p only specifically dimerizes those 60S subunits without apparent effect on the 80S ribosomes (Fig. 2F). The same experiment of Sdo1p on the 80S ribosomes was repeated in low Mg^2+^ condition (2.5 mmol/L). 80S ribosomes largely dissociate into 60S and 40S subunits in this dissociating condition (Fig. 2D), and the dissociated 60S subunits could be cross-linked by Sdo1p (Fig. 2E). However, in both of the high and low Mg^2+^ conditions, Sdo1p did not change the peak heights of the 80S ribosome (Fig. 2E and 2F), suggesting that the binding site of Sdo1p on the 60S subunit is highly likely at the inter-subunit face of the 60S subunit.

Based on the above observations, we conclude that Sdo1p preferentially binds to the 60S subunit, and is capable of promoting weak dimerization of mature 60S subunits. This reminds us of the recent observation that 80S ribosomes are capable of forming 80S-80S and 80S-60S dimers *in vivo* under amino acid depletion (Krokowski et al., 2011). Therefore, the 60S-60S dimer might also be a form of resting translation machinery induced by certain stresses, which appears to be in general consistent with previous reports showing that loss of SBDS in HEK293 or HeLa cells leads to increased sensitivity to various stresses (Ball et al., 2009; Watanabe et al., 2009).

### Domains I and II of Sdo1p are responsible for the 60S binding and dimerization

**Figure 3:**
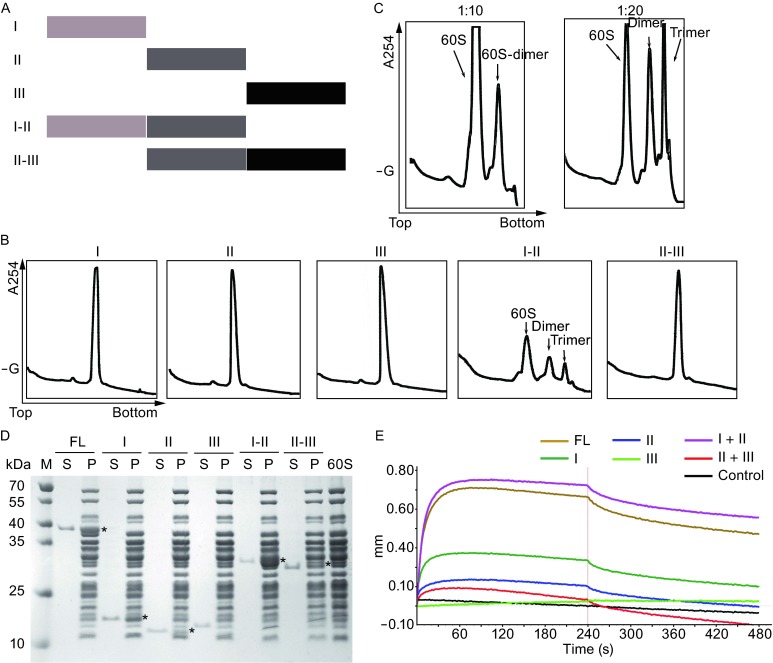
Sdo1p induced 60S dimerization through domain I-II. (A) Schematic overview of the Sdo1p variants. (B) Formation of 60S dimers by Sdo1p variants (20-fold excess) examined by SDGC. (C) The formation of 60S dimers by Sdo1p domains I-II is concentration dependent. (D) The binding of Sdo1p variants to the mature 60S subunit examined by co-sedimentation assay. FL, full-length; S, supernatant; P, pellet. (E) The binding of Sdo1p variants to the mature 60S subunit examined by Bio-layer interferometry. Both the association and dissociation reactions were carried out for 240 s

To map the domains of Sdo1p responsible for the binding and dimerization of the 60S subunit, we further characterized the binding of individual domains of Sdo1p to the 60S subunit. In human, SDS is an autosomal recessive disorder and mostly associated with two predominant mutations, 183-184TA to CT that results in an in-frame stop codon (K62X) and 258 + 2T to C that produces premature truncation (84Cfs3) (Boocock et al., 2003), both of which would render truncated forms of SBDS lack of C-terminal domains. Therefore, we constructed a series of Sdo1p truncations to test the ability of mutant proteins in the 60S binding and dimerizing abilities, including three individual domain constructs (I, II and III), and two truncations of terminal domains (I-II and II-III) (Fig. 3A).

Firstly, we tested the ability of these mutants in the 60S subunit dimerization by SDGC in the presence of chemical crosslinker. As shown in Fig. 3B, only the construct of domain I-II is capable of inducing the formation of 60S-60S dimer, and its dimerizing activity is also concentration-dependent (Fig. 3C). At a higher excess of domains I-II mutant (20-fold), a significant peak corresponding to trimers appears on the gradient profile. Next, we examined the binding of Sdo1p mutants to the 60S subunit by co-sedimentation assay. The reaction mixtures were subjected to a 33% sucrose cushion-based centrifugation and the binding was detected by Tricine-SDS-PAGE (Fig. 3D). Our results showed that similar to the full-length Sdo1p, constructs of domain I, II, and I-II all display clear binding to the 60S subunit. Very weak binding of construct II-III could also be detected. In contrast, no binding of domain III construct was detected. Lastly, in order to quantitatively compare the binding of these mutants to the 60S subunit, we measured the affinities of these mutants to the 60S subunit using Bio-layer interferometry (BLI) (Figs. 3E and S2). Highly consistent with the co-sedimentation data, construct of the domain I-II display the highest affinity to the 60S subunit (*K*_*D*_ = 15 nmol/L), comparable to the full-length protein (*K*_*D*_ = 18 nmol/L) (Fig. S2A and S2E). The truncated domain I, II and II-III display modest level of affinities (*K*_*D*_ ranging from 25–40 nmol/L) (Fig. S2B, S2C and S2F). In sharp contrast, domain III had no detectable binding (Fig. S2D).

From these results, a clear conclusion could be drawn: domain I of Sdo1p is the major RNA binding domain, while domain III has no RNA binding activity.

### 2D image analysis of the 60S-Sdo1p complexes

**Figure 4:**
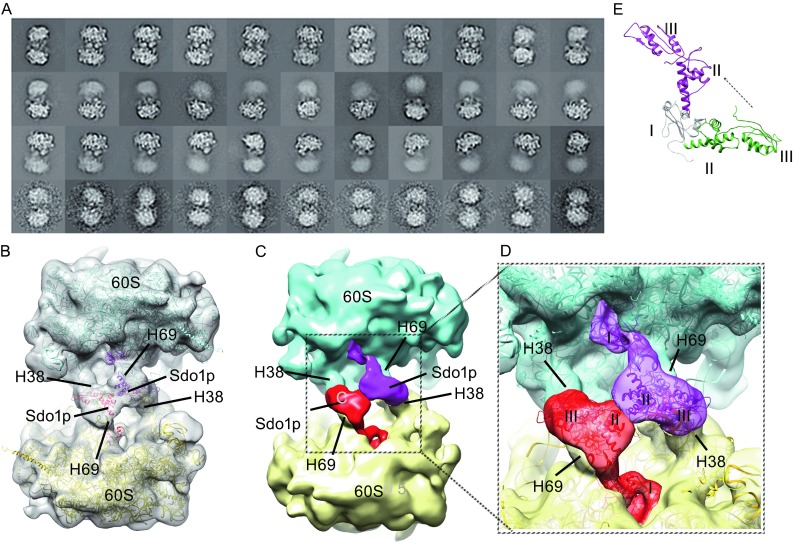
The cryo-EM structure of dimeric 60S-Sdo1p complex. (A) Reference-free 2D classification of selected particles of the 60S dimers. (B) Overview of cryo-EM density map of the dimeric 60S-Sdo1p complex, superimposed with fitted models. (C) Same as (B), but with two 60S subunits and two copies of Sdo1p separately colored. (D) Zoom-in view of boxed region in (C). (E) Comparison of Sdo1p in its 60S-bound conformation with that derived from homology modelling (see MATERIALS AND METHODS)

Knowing that different oligomeric states of the 60S-Sdo1p complexes could form, we processed cryo-EM images according to the particle sizes. Particles representing dimers were selected and subjected to reference-free alignment and classification by RELION (See MATERIALS AND METHODS). As shown in Fig. 4A, many populated class-average images show well resolved structural features for both 60S subunits, indicating that inter-subunit connection could be rather rigid (Fig. 4A). Interestingly, these 2D average images display an apparent 2-fold symmetry. This observation demonstrates that the dimer induced by Sdo1p is a specific structural entity, and not from random aggregation of 60S subunits or Sdo1p. Nevertheless, many average images have defined structural details only for one 60S subunit, with a smeared density blob for the other, suggesting that a certain extent of flexibility for the inter-subunit connection. A simple explanation for different orientations of two subunits within dimers is that Sdo1p and the 60S subunit could exist in different stoichiometry. In rigid dimers, the 60S subunit and Sdo1p are present in a 2:2 ratio, as two copies of Sdo1p would further “staple” two subunits to fix their relative orientation. In contrast, in loose dimers, one copy of Sdo1p is capable of bridging the two subunits, but the high inter-domain flexibility of Sdo1p (de Oliveira et al., 2010) would allow a wobbling between two subunits.

### 3D structure of the 60S-60S dimer

From 2D image analysis, the 60S subunit and Sdo1p could form stable 60S-60S dimers, which should enable the 3D structural determination of dimers by single particle analysis. Towards this end, different 3D classification strategies were tested on particles of dimers, and both the symmetry-free and 2-fold symmetric reconstructions were explored (Fig. S3). The 3D classification was done using RELION in a multi-round way. After the first round (C2-imposed), particles with 3D class structures showing relatively rigid dimers were kept for further analysis. Following steps were carried out without imposing two-fold symmetry. As a result, only 11% of particles displayed structural details for both 60S subunits, and structural refinement from these particles rendered a final density map at 14-Å resolution (Fig. S3B). This is expected, as 2D image analysis indicates that many dimers are not rigid dimmers (Fig. 4A). Using the crystal structure of the 60S subunit as template (Ben-Shem et al., 2011), additional densities between the two 60S subunits can be segmented (Fig. 4B–D). This mass of densities is sandwiched between H69 of one 60S subunit and H38 of the other 60S subunit.

The homology model of yeast Sdo1p was predicted by I-TASSER (Roy et al., 2010) and fitted into the cryo-EM density map (Fig. 4D). The additional densities between two 60S subunit allowed the docking of two copies of Sdo1p. Although the resolution of our map (14 Å) could not provide unambiguous assignment of individual domains into the density map, the relative orientation of individual domains on the 60S subunit could be fixed (Fig. 3) by integrating the very recent cryo-EM data of a chimerical 60S-SBDS complex (Weis et al., 2015), which revealed that domain I lies in the P-site. Very interestingly, the recent data also showed that domain II-III could exist in very different conformations, owing to the flexible linker between domain I and domain II (Weis et al., 2015). As a result, we built a model for the 60S-Sdo1p dimer (Fig. 4D), which requires a large reorientation of domain II-III (Fig. 4E), compared with the homology model of Sdo1p. The model could well explain the structural basis for the observed dimerization of 60S subunits by Sdo1p: domain I interacts with the ribosomal P-site of one subunit, while domains II-III (mostly II) interact with H38 of the other subunit.

### Interaction of Sdo1p with the 60S subunit is highly dynamic and might involve uL16

The relatively low population of the stable 60S dimers in cryo-EM images hampered the resolution of the dimer density map. We, therefore, sought to improve the structure by only processing the monomeric 60S particles. As a result, we prepared another batch of sample using 10-fold excess of Sdo1p in the 60S binding and only picked well-separated 60S particles from cryo-EM images. Although with a much larger dataset (305,370 particles), exhaustive 3D classification did not reveal high-resolution densities for Sdo1p. When more particles were included for final refinement, as expected, we could only obtain a high-resolution structure for the 60S subunit. In contrast, when a small homogenous fraction of particles were used, an additional piece of density could be seen in the ribosomal P-site. (Fig. S4). Based on our domain assignment in the 60S dimer, it is likely domain I of Sdo1p.

To further confirm our structural and biochemical data that Sdo1p binds to the ribosomal P-site, we employed cross-linking based mass spectrometry (CX-MS) (Singh et al., 2010), which is particularly useful for structural analysis of flexible and transient interactions. Therefore, we treated the 60S-Sdo1p complex with cross-linking reagents disuccinimidyl suberate (DSS) and bis(sulfosuccinimidyl) suberate (BS^3^), and subjected the linked sample to mass spectrometry. The reactive sites of the cross-linkers interact with the primary amino group of lysine and N-terminal tail of the protein. Considering the 11.4-Å spacer arm between reactive sites, lysine side-chains within 30 Å could be potentially cross-linked.

**Figure 5:**
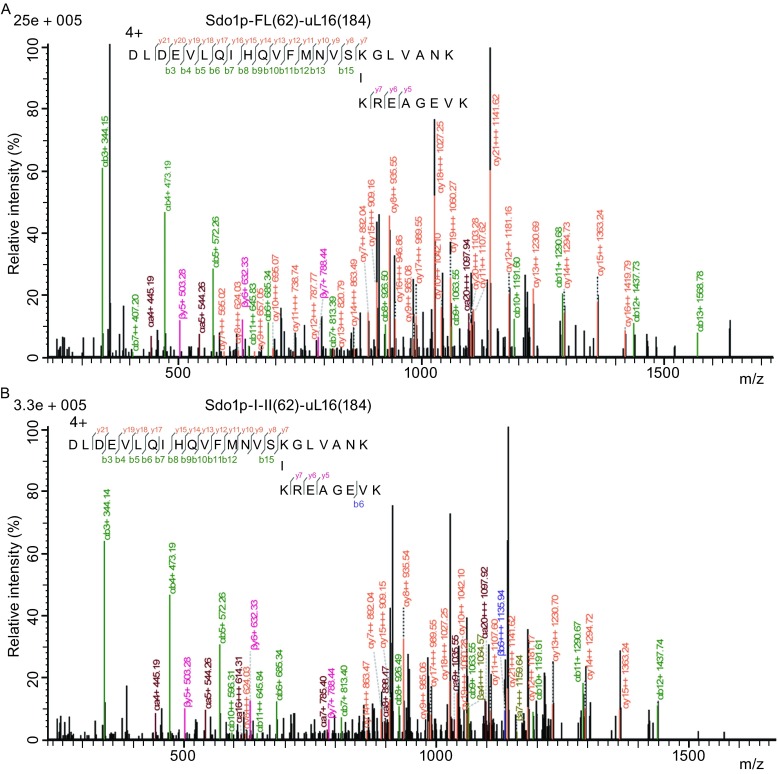
Crosslinking of Sdo1p with uL16 identified in CX-MS. (A) Representative MS spectra of the cross-linked peptides between Sdo1p and uL16 detected in the samples of the 60S-Sdo1p-FL (full-length) complex (A), the 60S-Sdo1p-I-II (Domain I-II) complex (B). Primary sequences of linked peptides are shown, with sites of cleavages labelled in different colors in both the sequences and spectra. 4+ or 3+ indicates the charge of the cross-linked peptides

We performed chemical cross-linking of the 60S complexes prepared with the full-length, domain I or domain I-II of Sdo1p. The CX-MS results show that a predominant cross-linking is between Sdo1p and uL16 through Lys62 (Sdo1p)-Lys184 (uL16) (Figs. 5A,  5B and S5), which is highly consistent with previous data that uL16 is involved in Sdo1p recruitment in the late-stage maturation of the 60S subunit (Sulima et al., 2014a). Another crosslinking was seen between Lys68 of Sdo1p and Lys of uL5 (Fig. S5).

Altogether, these results demonstrate that the interaction of Sdo1p with the 60S subunit is highly dynamic, which might be essential for its function in probing the conformational status of the ribosomal P-site during the late stage of cytoplasmic maturation of the 60S subunit.

## Discussion

**Figure 6:**
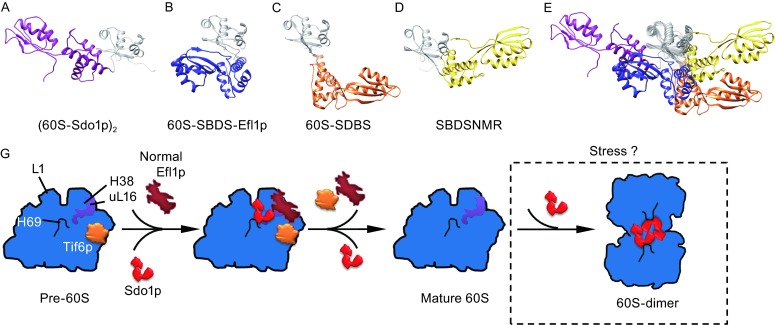
Proposed model of the action of Sdo1p in the late-stage maturation of the 60S subunit. (A–E) Conformational states of Sdo1p/SBDS. Coordinates of SBDS in heterologous 60S-SBDS-Efl1p (B) and 60S-SBDS (C) complexes were taken from a very recent cryo-EM work (Weis et al., 2015). One snapshot of NMR structures of human SBDS (D) was taken from a previous study (de Oliveira et al., 2010). All the structures were aligned using the domain I of Sdo1p/SBDS as reference. (G) In normal condition, Sdo1p, as a member of a conformational signal relay system, probes the maturation status of the ribosomal P-site, and passes the signal to Efl1p to release Tif6p from the nearly mature 60S subunit. Under a certain stress, Sdo1p might induce the formation of inactive 60S dimers to limit the cellular translation capacity

According to the established framework of Sdo1p in the 60S maturation, the function of Sdo1p is, by interacting with Efl1p, to promote the release of nucleolar shuttling factor Tif6p (Menne et al., 2007). Domains II-III of SBDS were reported to directly interact with Efl1p (Asano et al., 2014), and it was proposed that Sdo1p/SBDS might act as nucleotide exchange factor to stabilize the binding of GTP to Efl1p (Gijsbers et al., 2013). Because the binding site of Tif6p (Gartmann et al., 2010; Klinge et al., 2011) is rather distant from the binding site of Sdo1p on the 60S subunit, these data have put Sdo1p in a central position of this maturation pathway. Our data show that Sdo1p interacts with the ribosomal P-site (H69) through its domain I, with terminal domain II-III capable of moving around (Fig. 4E), suggesting that the essential role of Sdo1p is to probe the conformational status of the ribosomal P-site (Fig. 6G). Once the correct/native conformation of the ribosomal P-site is established, which in turn stabilizes domain II-III of Sdo1p to induce Tif6p-releasing activity of Efl1p. This hypothesis is further supported by the essential role of uL16 in the maturation of the 60S subunit. The uL16 was also found to cooperate with Sdo1p and Efl1p to release Tif6p, as well as Nmd3 (Bussiere et al., 2012; Hedges et al., 2005; Menne et al., 2007; Sulima et al., 2014a; Sulima et al., 2014b; West et al., 2005). More importantly, uL16 (R98S) mutant in yeast causes a defect in late-stage 60S subunit maturation and targets mutant ribosomes for degradation (Sulima et al., 2014b), suggesting a checkpoint role of uL16 in the quality control of the 60S maturation (Sulima et al., 2014a). Our CX-MS data indicate that Sdo1p (domain I) is in close contact with uL16. Taking into consideration of the critical role of the P-site loop of uL16 in Sdo1p binding (Sulima et al., 2014a) and Tif6p release (Bussiere et al., 2012), it is plausible that Sdo1p senses the incorporation and conformational maturation of uL16 on the 60S subunit and pass the signal to Efl1p. Also, our structural data reveal that H69 and H38 are two binding partners of Sdo1p, both of which are highly conserved (structurally and functionally) in eukaryotes and prokaryotes. And these two helices are known to be highly dynamic and adopt very different conformations in the pre-60S (yeast) (Bradatsch et al., 2012; Greber et al., 2012; Leidig et al., 2014) or pre-50S (bacterial) (Jomaa et al., 2014; Li et al., 2013; Zhang et al., 2014a) particles. Taken together, our data support a conformational signal relay system for these factors (uL16-Sdo1p-Efl1p-Tif6p), in which Sdol1p and Efl1p couple two spatially distant events during the late-stage maturation of the 60S subunit, the ribosomal P-site maturation and the release of anti-association factor Tif6p (Gartmann et al., 2010). The dynamic nature of Sdo1p on the 60S subunit ensures its participation as a probing factor to sense the conformational maturation of its surrounding rRNA and RP.

Our structural data suggest that Sdo1p remains very dynamic on the 60S subunit. Very interestingly, in comparison with the very recent data on chimerical 60S-SBDS and 60S-SBDS-Efl1p (Weis et al., 2015), domains II-III of Sdo1p/SBDS could adopt very different conformations on the 60S subunit (Fig. 6A–E). These observations are consistent with the NMR spectroscopic analysis of SBDS in solution (de Oliveira et al., 2010). Altogether, these results support our hypothesis that Sdo1p is a dynamic probe of the maturation status of the 60S subunit, and the inter-domain flexibility of Sdo1p/SBDS is essential for its function.

Our CX-MS data also indicate a proximity for uL5 and Sdo1p. This observation might be of physiological significance to the disease of SDS in human. It was generally believed that partial loss of SBDS function in SDS might induce a nucleolar stress from defective ribosome biogenesis and activate the RP (ribosomal protein)-p53-HDM2 pathway, and uL5 (RPL11) is one of RPs involved in stabilizing p53 following nucleolar stress (Deisenroth and Zhang, 2010; Holmberg Olausson et al., 2012; Nakhoul et al., 2014).

In the present work, we found that Sdo1p could induce the formation of 60S-60S dimers *in vitro* through crosslinking H69 and H38 from the two participating 60S subunits. In bacterial and mammalian cells, it was shown that higher-order organization of ribosomes is employed by the cells as a means to regulate the translation capacity by limiting the number of active ribosomes. In *E. coli*, upon transition to stationary phase, cells start to accumulate 100S particles (Yoshida and Wada, 2014), composed of 70S dimers induced by protein factors called ribosome modulation factor (RMF) (Wada et al., 1995) and hibernation promoting factor (HPF) (Ueta et al., 2005). When mammalian (rat) cells were challenged by amino acid starvation, a form of 110S particles, including 80S dimers and 80S-60S heterodimers, were also detected (Krokowski et al., 2011). These previous data indicate a conserved mechanism of translation modulation by organizing ribosomes into resting higher-order assemblies. Previous work also suggested SBDS has a second role in multiple cellular stress response pathways, independent of its primary role in ribosome biogenesis (Ball et al., 2009). HEK293 cells with the deletion of SBDS are hypersensitive to DNA damage and endoplasmic reticulum stress (Ball et al., 2009). HeLa cells with SBDS-knockdown, as well as SDS patient cells are hypersensitive to Fas-mediated apoptosis (Watanabe et al., 2009; Watanabe and Dror, 2005). In yeast, Sdo1p physically interacts with Btn1p (CLN3 in human) and was suggested to involved in cellular responses to pH and nutrient changes (Vitiello et al., 2010). Therefore, it is possible that the 60S dimers induced by Sdo1p, if not an *in vitro* artifact, might represent a form of inactive storage of ribosomal subunits upon a certain stress (Fig. 6G). This attractive hypothesis merits further investigation.

## Materials and methods

### Gene cloning and protein purification

The genes encoding full-length and truncated Sdo1p were amplified using standard Polymerase Chain Reaction (PCR) from *S. cerevisiae*. Primers were designed according to respective domains of yeast Sdo1p to clone the full-length (1–250), domain I (1–95), domain II (96–169), domain III (170–250), domain I-II (1–169) and domain II-III (96–250) constructs. The fragments were cloned into pET21b vector to yield pET21b-Sdo1p constructs with C-terminal 6× -His-tag for purification. The plasmids were transformed into *E. coli* BL21 (DE3) cells for overexpression.

For protein purification, cells were cultured in LB media containing 50 μg/mL of ampicillin at 30°C to an OD_600_ of 1.0 and induced by 0.5 mmol/L IPTG for 4 h. Cells were pelleted and suspended in buffer A (50 mmol/L Tris-HCl [pH 7.5], 500 mmol/L NaCl, 1 mmol/L PMSF) containing 30 mmol/L imidazole and disrupted by an ultrasonic processor. Cell lysates were clarified by centrifugation at 13,000 rpm (Avanti J-26 XP, JA25.50 rotor, Beckman Coulter) for 1 h. The clarified lysates were load onto a Ni-NTA column (GE Healthcare) and eluted with buffer B (same as buffer A but containing 500 mmol/L imidazole). The target proteins were pooled, concentrated with Millipore Amicon Ultra Centrifugal filters and subjected to gel filtration chromatography (Superdex75 10/300 GL, GE Healthcare) with buffer C (25 mmol/L Tris-HCl [pH7.5], 100 mmol/L KCl, 2.5 mmol/L MgCl_2_). Purified proteins were split into aliquots and stored at −80°C.

### Ribosomal subunit purification

Yeast cells (*S. cerevisiae* S288C) were grown in 3 L of YEPD (1% yeast extract, 2% Bacto peptone and 2% glucose) medium to an OD_600_ of 1.0. Ribosome purification was carried out as previously described (Zhang et al., 2014b). Cells were harvested and washed twice with ice-cold Lysis buffer (50 mmol/L Tris-acetate [pH7.0], 50 mmol/L NH_4_Cl, 12 mmol/L MgCl_2_, 1 mmol/L DTT, 1 pill/50-mL complete protease inhibitor cocktail [Roche]). Resuspended cells were disrupted with a high-pressure homogenizer (5000 p.s.i.) for three times. Cell lysates were centrifuged at 13,000 rpm for 1 h at 4°C in a JA 25.50 motor (Beckman Coulter). Supernatants were layered over a 10%–50% linear sucrose gradient (50 mmol/L Tris-acetate [pH 7.0], 100 mmol/L NH_4_Cl, 12 mmol/L MgCl_2_, 1 mmol/L DTT), and centrifuged at 30,000 rpm for 5 h in an SW32 rotor (Beckman Coulter) at 4°C. The gradient profile was monitored at 254-nm wavelength and fractionated in a gradient collector (Teledyne Isco). Fractions of 80S ribosomes were collected, concentrated with Amicon Ultra Centrifugal filters (Millipore) and washed with separation buffer (50 mmol/L Tris-HCl [pH 7.4], 500 mmol/L KCl, 2.5 mmol/L MgCl_2_, 1 mmol/L DTT). After the buffer was changed to separation buffer, the mixture was incubated at 37°C for 10 min. Reaction mixtures were layered over a 10%–40% linear sucrose gradient (50 mmol/L Tris-HCl [pH 7.4], 500 mmol/L KCl, 2.5 mmol/L MgCl_2_, 1 mmol/L DTT) and centrifuged at 30,000 rpm in an SW32 rotor (Beckman Coulter) at 4°C for 7 h. The gradient was similar analyzed and fractions of 40S and 60S subunits were separately collected. After concentrating with Millipore Amicon Ultra Centrifugal filters, samples were split into aliquots and stored at −80°C for further use.

### *In vitro* binding with gel filtration

The 60S subunit (150 pmol) and full-length Sdo1p (20-fold excess) were mixed in 500-µL buffer C (25 mmol/L Tris-HCl [pH 7.5], 100 mmol/L KCl, 2.5 mmol/L MgCl_2_), and incubated at 30°C for 15 min. The mixture was loaded onto a gel filtration column (Superose6 10/300 GL, GE Healthcare). The peak fractions were subjected to SDS-PAGE.

### Co-sedimentation assay

60S ribosomal subunits 50 pmol) were incubated with full-length or truncated Sdo1p proteins in a ratio of 1:20 for 15 min at 30°C in 90 µL buffer. The mixtures were then loaded onto a sucrose cushion (25 mmol/L Tris-HCl [pH 7.5], 100 mmol/L KCl, 2.5 mmol/L MgCl_2_, 33% sucrose) and centrifuged at 95,000 rpm for 2 h in a TLA-100 rotor (Beckman Coulter) at 4°C. The supernatants (1/18) and the pellets (1/3) were collected separately and resolved by Tricine-SDS-PAGE.

### Sucrose density gradient centrifugation (SDGC)

Samples of ribosomes and Sdo1p variants were changed to a Hepes-KOH buffer system (25 mmol/L Hepes-KOH [pH7.5], 100 mmol/L KCl, 2.5 mmol/L MgCl_2_). 37.5 pmol ribosomes (40S, 60S or 80S) were incubated with full-length or truncated Sdo1p proteins in a ratio of 1:20 for 15 min at 30°C in 200 µL buffer. The mixtures were incubated with or without 0.1% glutaraldehyde for another 15 min, layered onto a 10%–50% linear sucrose gradient (25 mmol/L Hepes-KOH [pH 7.5], 100 mmol/L KCl, 2.5 mmol/L MgCl_2_), and centrifuged at 39,000 rpm for 4 h in an SW41 rotor (Beckman Coulter) at 4°C. The gradient was analyzed and fractionated in an ISCO gradient collector.

### *In vitro* binding with Bio-layer Interferometry

The BLI-based experiments were performed with Octet RED96 System (ForteBio, Pall Corp.) according to the manufacturer’s instructions. Sdo1p variants (wild type and mutants) in Hepes-KOH buffer system (25 mmol/L Hepes-KOH [pH 7.5], 100 mmol/L KCl, 2.5 mmol/L MgCl_2_) were immobilized to the optic biosensors of Anti-Penta-HIS (HIS1 K, ForteBio). Before each experiment, biosensors were pre-equilibrated with binding buffer, followed by equilibrium binding with Sdo1p variants (2 μg/mL). Pre-experiments were carried to determine the optimal concentration range of the 60S subunit suitable for the measurements. For the single concentration experiments, a final concentration of 48 nmol/L 60S subunit was used. For the concentration gradient experiments, 2-fold dilution series of the 60S subunit were used (48 nmol/L, 24 nmol/L, 12 nmol/L, 6 nmol/L, 3 nmol/L, and 1.75 nmol/L). Both the association and dissociation of each measurement were carried out for 240 s. The data was analyzed and dissociation constants were calculated by the software of the Data analysis 7.0 provided by ForteBio.

### Cryo-sample preparation and data collection

The 60S subunit (100 nmol/L) was incubated with 20-fold or 10-fold excess of full-length Sdo1p at 30°C for 15 min. The mixture was diluted to a final concentration of 60 nmol/L for the 60S complex in binding buffer. 4-µL aliquots of samples were applied to 300-mesh 2/2 glow-discharged Quantifoil grids (Quantifoil Micro Tools) which were pre-coated with a thin layer of carbon. The grids were blotted and plunged into liquid ethane with an FEI Mark IV Vitrobot operated at 4°C. For the sample of dimeric 60S-Sdo1p complexes (20-fold excess of Sdo1p), data collection was performed with an FEI F20 at 80,000× magnification with a Gatan UltraScan 4000 CCD camera. For the sample of monomeric 60S-Sdo1p complexes, data collection was done with an FEI Titan Krios equipped with an FEI Eagle 4 K × 4 K CCD camera at 75,000× magnification. All the images were recorded under low-dose conditions (~20 e-/Å^2^) with AutoEMation package (Lei and Frank, 2005).

### Image processing and structural analysis

Micrograph screening, estimation of contrast transfer function parameters, and initial particle picking were performed with SPIDER package (Shaikh et al., 2008). An artificial 60S dimer was used as the template for automatic particle picking (Rath and Frank, 2004). For 60S-Sdo1p dimers, 116,271 particles (from 3,294 micrographs) (2.76 Å/pixel with a binning factor of two) were picked and subjected reference-free 2D classification using XMIPP (Scheres et al., 2008), EMAN2 (Tang et al., 2007) and RELION (Scheres, 2012) packages, which rendered essentially similar results with many of the dimers displaying considerable flexibility on the orientation of the two 60S subunits. For the 3D classification, all the particles were classified into 6 classes without or with C2 symmetry imposed using RELION package. For C2-imposed classification, two classes display reliable details for both two 60S subunits were combined (30,452 particles) and subject to another round of 3D classification. The second round was done without imposing the C2-symmetry, resulting into 4 classes. Two of them were combined (13,219 in total) and subjected to structural refinement by RELION. The final resolution of the refined map is 14 Å based on the 0.143 cutoff criteria of the gold-standard Fourier shell correlation (FSC).

For monomeric 60S-Sdo1p complexes, 305,371 particles (2.33 Å/pixel with a binning factor of two) (from 7,994 micrographs) were classified into 10 classes using RELION. One class (40,186 particles) displayed substantial additional density at the ribosomal P-site. After structural refinement, the final reported resolution is 9 Å based on the 0.143 cutoff criteria of the gold-standard FSC.

The yeast homology model of Sdo1p was modelled using I-TASSER (Roy et al., 2010). The fitting of two copies of Sdo1p was done manually using UCSF Chimera (Pettersen et al., 2004), and optimized by the “fit in map” module of Chimera. Coordinates of the yeast ribosome were from a previous crystallography study (Ben-Shem et al., 2011).

### Cross-linking mass spectrometry analysis (CX-MS)

50 pmol 60S ribosome was incubated with full-length or truncated Sdo1p at 1:20 molar ratio for 15 min at 30°C (25 mmol/L Hepes-KOH [pH 7.5], 100 mmol/L KCl, 2.5 mmol/L MgCl_2_). The mixture was cross-linked with DSS or BS3 (Thermo Fisher Scientific) at 1:1 mass ratio at room temperature for 1 h. The reaction was then quenched with 20 mmol/L ammonium bicarbonate. Cross-linking products were analyzed by SDS-PAGE to evaluate the cross-linking efficiency. For MS analysis, proteins were precipitated with acetone, resuspended in 8 mol/L urea, 100 mmol/L Tris, pH 8.5, and digested with trypsin. LC-MS/MS analyses were carried on an EASY-nLC 1000 system (Thermo Fisher Scientific) interfaced with a Q-Exactive mass spectrometer (Thermo Fisher Scientific). Peptides were separated on an analytical capillary column (75 μm × 10 cm, 1.8 μm C18) using a 60 min linear gradient at a flow rate of 200 nL/min. The mass spectrometer was operated in data-dependent mode with one full MS scan followed by ten HCD MS/MS scans with a dynamic exclusion time of 30 s. Precursors of the +1, +2, or unassigned charge states were rejected. pLink (Yang et al., 2012) was used for identification of cross-linked peptides by requiring FDR <5%.

## Accession code

The density map of the 60S-Sdo1p dimer has been deposited in the EMdataBank under accession codes of EMD-3280.

## Electronic supplementary material

Supplementary material 1 (PDF 605 kb)
