# Supplementary material for: Structural dynamics of the yeast Shwachman-Diamond syndrome protein (Sdo1) on the ribosome and its implication in the 60S subunit maturation
Source: Protein Cell. 2016 Feb 5;7(3):187–200. doi: 10.1007/s13238-015-0242-5 (PMC4791427; doi:10.1007/s13238-015-0242-5)
Supplement: Supplementary file 1 — Supplementary material 1 (PDF 605 kb) [file 13238_2015_242_MOESM1_ESM.pdf]

Supplemental data

Structural dynamics of the yeast Shwachman-Diamond syndrome protein (Sdo1) on theribosome and its implication in the 60S subunit maturation

Chengying Ma[1], Kaige Yan[1], Dan Tan[2,3], Ningning Li[1], Yixiao Zhang[1], Yi Yuan[1], Zhifei Li[1], Meng-Qiu Dong[2,3], Jianlin Lei[1] and Ning Gao[1,4]

[1]School of Life Sciences, Tsinghua University, Beijing, Beijing 100084, China

[2]National Institute of Biological Sciences, Beijing, Beijing 102206, China

[3]Graduate Program in Chinese Academy of Medical Sciences and Peking Union Medical College, Beijing 100730, China

[4]To whom correspondence may be addressed: School of Life Sciences, Tsinghua University, Beijing 100084, China. Tel: +86-10-62794277; E-mail: ninggao@tsinghua.edu.cn.

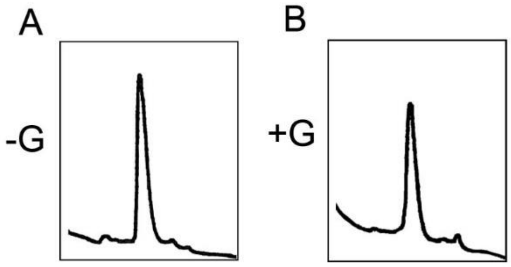

Supplementary figure S1. 0.1% Glutaraldehyde does not induce 60S dimerization.

60S subunits were applied to sucrose density gradient centrifugation in the absence (-G) (A) or presence (+G) (B) of 0.1% glutaraldehyde.

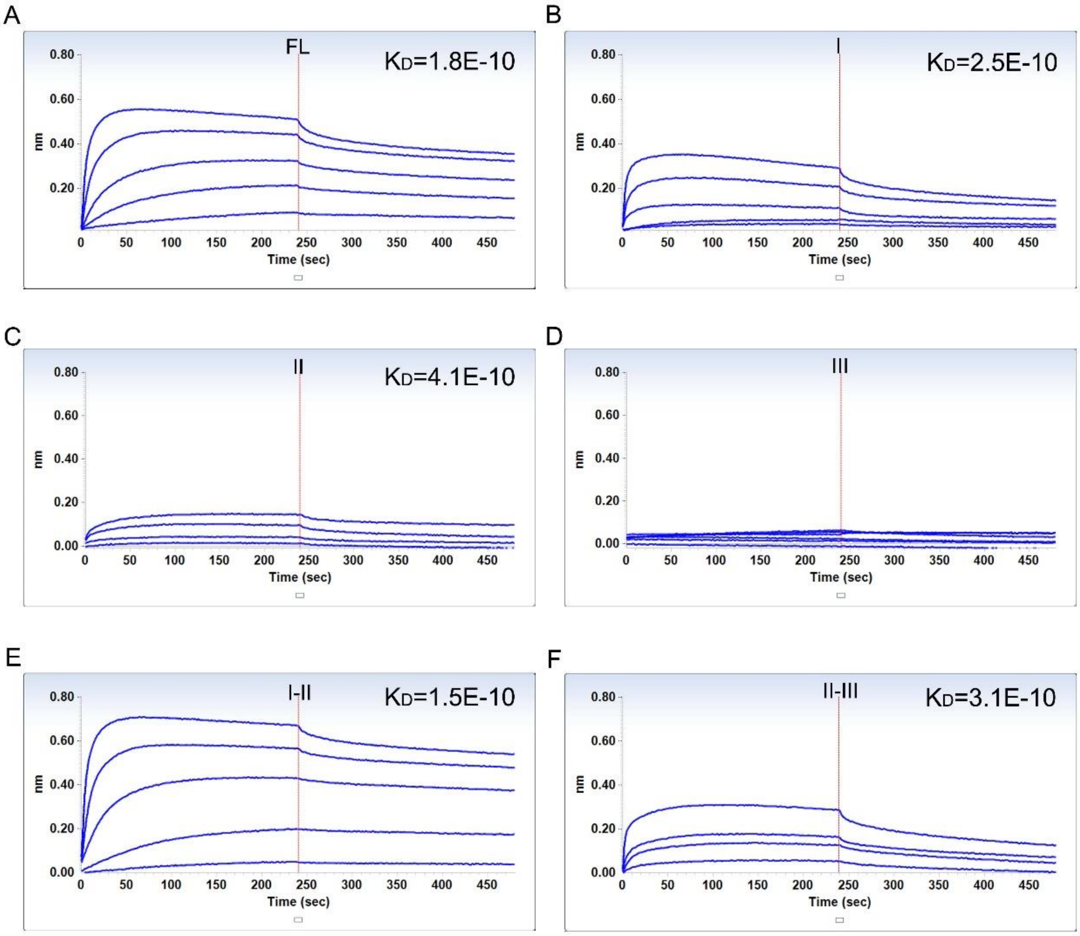

Supplementary figure S2. Binding of Sdo1p variants to the 60S subunit examined by Bio-layer interferometry.

(A-F) Global kinetic analysis of the binding of Sdo1p variants to the 60S subunit. Both association (240 sec) and dissociation (240 sec) curves for the binding of FL (full-length), I (domain I), II (domain II), III (domain III), I-II and II-III mutants of Sdo1p to the 60S subunit are shown. Calculated K_D was also labelled.

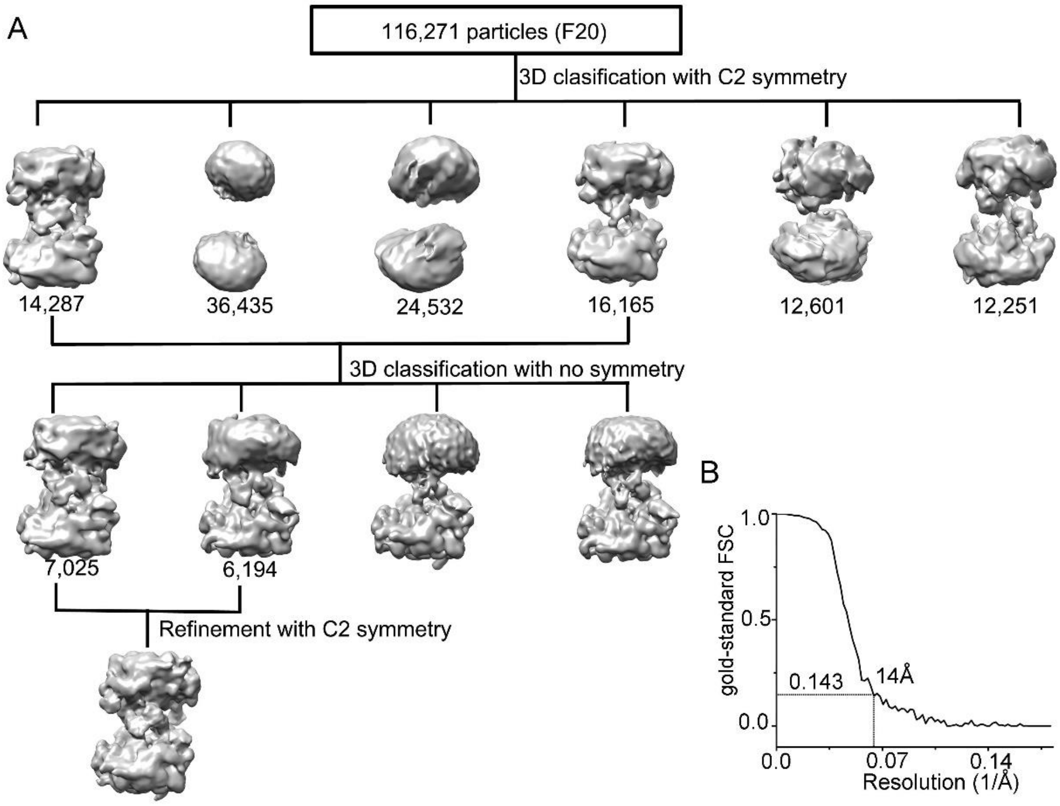

Supplementary figure S3. 3D classification of the dimeric 60S-Sdo1p particles

(A) A total of 116,271 particles were picked and subjected to a cascade of 3D classification. The first round was done with C2-symmetry imposed. Two of the resulting 6 classes were combined and subject to another round of symmetry-free 3D classification. A final dataset (13,219, 11% particles) containing particles from two classes of the second 3D classification was subjected to structural refinement with C2-symmetry imposed. (B) Gold-standard Fourier shell correlation (FSC) curve of the final cryo-EM density map.

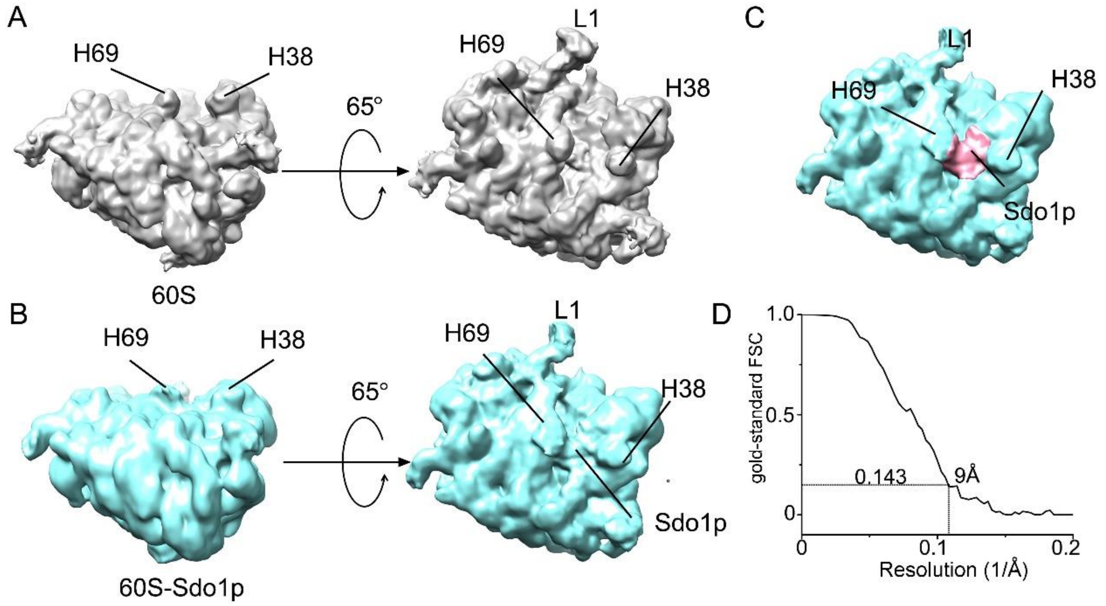

Supplementary figure S4. Structural comparison of the monomeric 60S-Sdo1p complex with the empty 60S subunit.

(A) Two representative views of the empty 60S subunit. (B) Same as (A), but of the monomeric 60S-Sdo1p complex. (C) Cryo-EM density map of the monomeric 60S-Sdo1p complex, with highlight of the additional density at the ribosomal P-site (red), which could be attributed to Sdo1p (likely domain I). (D) Gold-standard Fourier shell correlation (FSC) curve of the final cryo-EM density map.

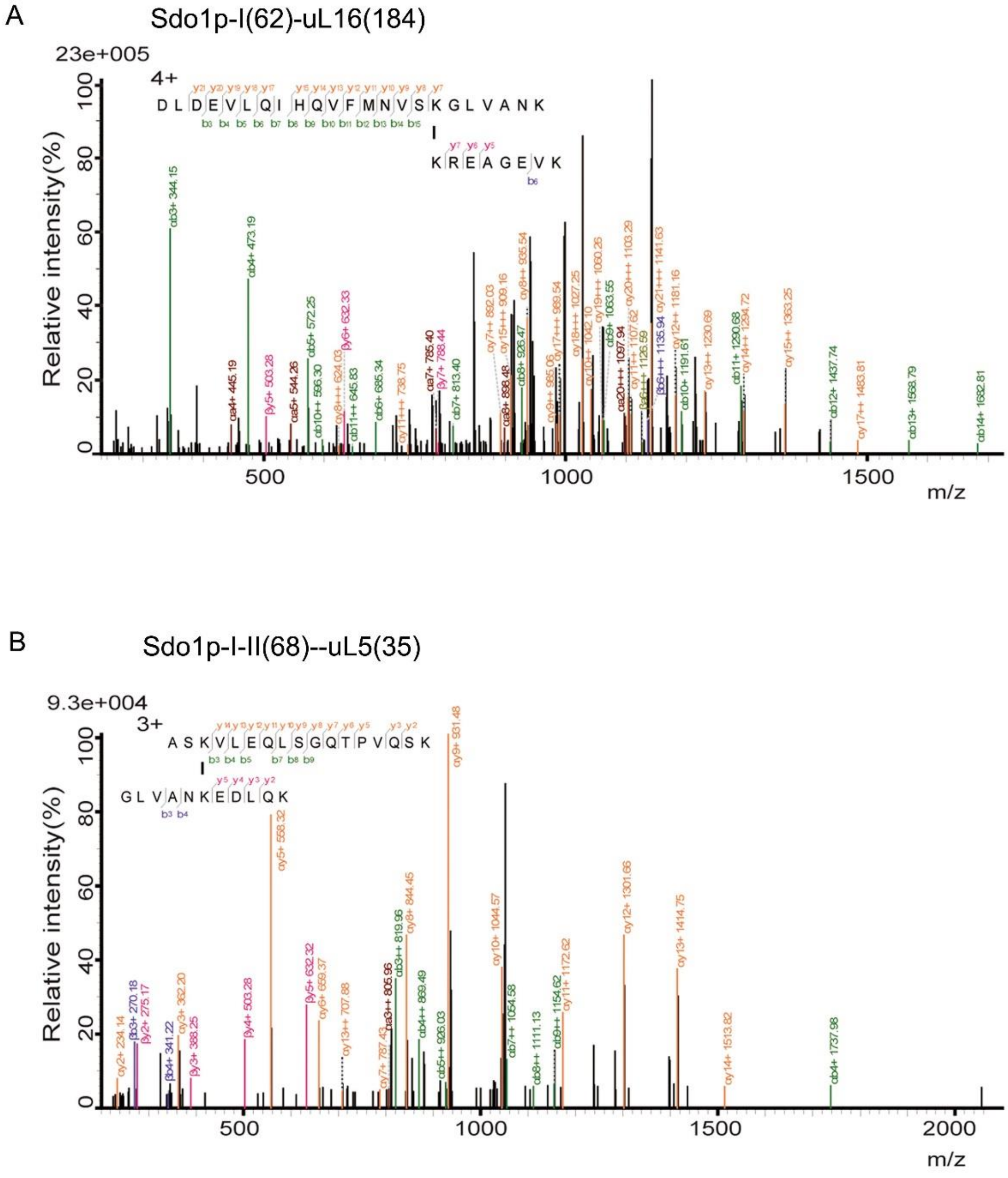

Supplementary figure S5. CX-MS analysis of 60S-Sdo1p complexes.

(A) Representative MS spectra of the cross-linked peptides between Sdo1p and uL16 detected in the samples of the 60S-Sdo1p-I (Domain I) complex. (B) Crosslinked peptides between Sdo1p and uL5 detected in the samples of the 60S-Sdo1p-I. Primary sequences of linked peptides are shown, with sites of cleavages labelled in different colors in both the sequences and spectra. 4+ or 3+ indicates the charge of the cross-linked peptides.
